# Pelvic target volume inter-fractional motion during radiotherapy for cervical cancer with daily iterative cone beam computed tomography

**DOI:** 10.1186/s13014-024-02438-1

**Published:** 2024-04-15

**Authors:** Zheng Zeng, Jiawei Zhu, Zhiqun Wang, Guangyu Wang, Junfang Yan, Fuquan Zhang

**Affiliations:** 1 Department of Radiation Oncology, Peking Union Medical College Hospital, Chinese Academy of Medical Sciences & Peking Union Medical College, 100730 Beijing, China; 2 Department of Radiation Oncology, State Key Laboratory of Complex Severe and Rare Diseases, Peking Union Medical College Hospital, Chinese Academy of Medical Science and Peking Union Medical College, 100730 Beijing, China

**Keywords:** Cervical cancer, Clinical target volume, Inter-fractional motion, Radiotherapy

## Abstract

**Background:**

Tumor regression and organ movements indicate that a large margin is used to ensure target volume coverage during radiotherapy. This study aimed to quantify inter-fractional movements of the uterus and cervix in patients with cervical cancer undergoing radiotherapy and to evaluate the clinical target volume (CTV) coverage.

**Methods:**

This study analyzed 303 iterative cone beam computed tomography (iCBCT) scans from 15 cervical cancer patients undergoing external beam radiotherapy. CTVs of the uterus (CTV-U) and cervix (CTV-C) contours were delineated based on each iCBCT image. CTV-U encompassed the uterus, while CTV-C included the cervix, vagina, and adjacent parametrial regions. Compared with the planning CTV, the movement of CTV-U and CTV-C in the anterior-posterior, superior-inferior, and lateral directions between iCBCT scans was measured. Uniform expansions were applied to the planning CTV to assess target coverage.

**Results:**

The motion (mean ± standard deviation) in the CTV-U position was 8.3 ± 4.1 mm in the left, 9.8 ± 4.4 mm in the right, 12.6 ± 4.0 mm in the anterior, 8.8 ± 5.1 mm in the posterior, 5.7 ± 5.4 mm in the superior, and 3.0 ± 3.2 mm in the inferior direction. The mean CTV-C displacement was 7.3 ± 3.2 mm in the left, 8.6 ± 3.8 mm in the right, 9.0 ± 6.1 mm in the anterior, 8.4 ± 3.6 mm in the posterior, 5.0 ± 5.0 mm in the superior, and 3.0 ± 2.5 mm in the inferior direction. Compared with the other tumor (T) stages, CTV-U and CTV-C motion in stage T1 was larger. A uniform CTV planning treatment volume margin of 15 mm failed to encompass the CTV-U and CTV-C in 11.1% and 2.2% of all fractions, respectively. The mean volume change of CTV-U and CTV-C were 150% and 51%, respectively, compared with the planning CTV.

**Conclusions:**

Movements of the uterine corpus are larger than those of the cervix. The likelihood of missing the CTV is significantly increased due to inter-fractional motion when utilizing traditional planning margins. Early T stage may require larger margins. Personal radiotherapy margining is needed to improve treatment accuracy.

## Background

Cervical cancer is one of the most common malignant tumors of the female reproductive system [1]. Radiation therapy (RT) plays a major role in the treatment of cervical cancer [2]. The development of intensity-modulated radiation therapy (IMRT) can improve target conformity and reduce radiation exposure to organs at risk (OARs), such as the bladder, bowel, and rectum [3]. The position of the cervix and uterus maximal moves at 4–6 cm/day [4]. Meanwhile, changes in bladder and rectum filling have a profound influence on the shape and position of the cervix and uterus [5]. In addition, several studies have found that the uterus and cervix can move due to tumor regression [6, 7]. Considering these factors, a large margin is used to ensure a large enough target volume coverage during RT [8]. Movement of the uterus and cervix may result in inadequate target coverage, which may compromise tumor control [9].

Understanding the position variation of OARs and the target volume is essential before implementing IMRT. A 15 mm uniform margin was found to be effective in encompassing 68% of the clinical target volume (CTV), with significant interpatient variation [9]. A uniform planning margin would result in excessive irradiation to normal tissue, reducing the benefits of IMRT [10]. Different planning margins around subregions of the cervix and uterus is one approach to overcoming the variation.

In this study, we analyzed the inter-fractional movements of the uterus and cervix in cervical cancer patients with daily iterative cone-beam computed tomography (iCBCT) who underwent RT. Our findings will help clinicians to better guide radiation strategies for cervical cancer.

## Methods

### Patients

In this study, we analyzed 15 patients with stages IB–IVA cervical cancer treated with volumetric-modulated arc therapy (VMAT) at the Peking Union Medical College Hospital. The patients were staged according to the International Federation of Gynecology and Obstetrics (FIGO) and the American Joint Committee on Cancer TNM Staging System for cervical cancer. All patients were confirmed squamous cell cervical cancer by pathology. The detailed clinical characteristics are summarized in Table 1. Approval was obtained from the Institutional Review Board of Peking Union Medical College Hospital.

**Table 1:** Clinical characteristics of patients

Characteristics	No. (%)
Age (years old)	
Median	57
Range	48–70
Tumor size (cm)	
< 4 cm	6(40.0%)
≥ 4 cm	9(60.0%)
FIGO stage	
IB	3(20.0%)
IIA	3(20.0%)
IIB	3(20.0%)
IIIA	0
IIIB	3(20.0%)
IIIC	1(6.7%)
IVA	2(13.3%)
T stage	
T1	3(20.0%)
T2	7(46.7%)
T3	3(20.0%)
T4	2(13.3%)

Abbreviations: FIGO, International Federation of Gynecology and Obstetrics; T, tumor

### Simulation

Our previous studies have provided a comprehensive description of the simulation [2, 11]. Patients underwent simulation in the supine position with low-temperature thermoplastic masks using the 16-slice Philips Brilliance Big Bore CT scanner with 5 mm slice thickness. Before the simulation, patients were advised to ensure their bladder contained approximately 300–500 ml of urine and to empty their rectum. They were to take 10 ml of meglumine diatrizoate mixed with 200–300 ml of water, which was consumed 1.5 h before the simulation. Those with sufficient renal function received intravenous contrast.

### Treatment planning

Target determination was based on the imaging and clinical findings, as previously described [12]. All patients in this study received pelvic of external beam radiation therapy (EBRT). The radiotherapy dose was delivered to plan target volume (PTV) by VMAT. The prescribed dose of 50.4 Gy in 28 fractions using 6MV-X rays. For patients with lymph nodes metastasis, the dosage was increased to 56–60.2 Gy with simultaneous integrated boost.

### iCBCT

Patients were required to prepare the rectum and bladder, similar to the simulation. Prior to each treatment, daily iCBCT scans (Varian, Halcyon v. 2.0) were conducted to achieve rigid fusion and ensure proper couch alignment through bony match in three dimensions: left-right (LR), anterior-posterior (AP), and superior-inferior (SI). These iCBCTs were also utilized to monitor target coverage filling. The experienced clinicians assessed whether the PTV contained the primary tumor, uterus, cervix, vagina, and lymphatic drainage areas. If these structures were not positioned correctly within the PTV due to inadequate rectal or bladder preparation, the patient was instructed to perform rectal or bladder preparation once more. iCBCT was performed again, as described above. Once an acceptable alignment was achieved, RT was commenced.

### iCBCT image target delineation

According to previous studies [13], a bladder volume less than or equal to 30% compared to the volume of planning CT images was analyzed. The new CTVs were delineated by a radiation oncologist based on each iCBCT image. Two experienced radiologists specializing in gynecologic oncology reviewed all delineated target volumes. The cervix and uterus were also contoured separately at the uterine isthmus on each iCBCT. The clinical target volume of the uterus (CTV-U) included the uterus, while the clinical target volume of the cervix (CTV-C) included the cervix, vagina, and adjacent parametrial regions. All CTV-U and CTV-C iCBCT images were projected back to the planning CT, resulting in the ultimate CTV-U and CTV-C. In the planning CT, target coverage and inter-fractional motion were assessed, comparing initial CTV-C and CTV-U.

### Target coverage evaluation

A Python program for the automated analysis of the size of the inter-fractional internal target volume (ITV) during RT was designed using the Eclipse treatment planning system. The program processed iCBCT images and performed smoothing to avoid anomalies caused by small points or non-smooth contours during target delineation. The analysis was performed slice by slice in the anterior, posterior, rotational, and lateral directions with uniform expansion intervals of 0.1 cm. For the AP and LR direction, the program iteratively expanded the contours until the expanded PTV included the ITV. This process was used to obtain the motion in the AP and LR directions. In the SI direction, the program expanded in 0.5 cm intervals to obtain the motion. This resulted in a comprehensive assessment of all motions in different directions for the CTV. To further refine the evaluation, the PTV was contracted in the AP and LR directions, which stopped when it was near the ITV in a particular direction. The contraction continued in the other directions until it completely enclosed the ITV. This method was used to obtain the data sets, and the program automatically calculated the minimum expansion value that encompasses the ITV.

### Planning margins evaluation

Uniform three-dimensional planning margins of 3, 5, 7, 10, 15, 20, 25, and 30 mm were added to the CTVs from the planning CT to generate theoretical PTVs, which were analyzed target coverage with CTV-Us and CTV-Cs. For each CTV-PTV margin expansion, the assessed endpoints included the percentage of patients in whom CTV-U and CTV-C were not fully encompassed by the PTV.

### Statistical analysis

The mean, median, range and standard deviation of the magnitude of displacement of the target volume in each plane were calculated. All statistical analysis and processing were conducted using SPSS software, version 23.0 (IBM Corp, Armonk, NY, USA). Two-sided *P*-values < *0.05* were considered statistically significant. In cases where the data adhered to the normal distribution, the paired t-test was used for statistical analysis; alternatively, the Wilcoxon rank sum test was utilized when normality assumptions were not met.

## Results

### Number of iCBCT scans

Twenty-eight iCBCT scans were scheduled and attempted for each patient. A total of 420 iCBCT scans were obtained from 15 patients; however, some iCBCTs could not be delineated for target volume and evaluated due to artifacts or technical issues, such as excessive intestinal gas, large respiratory amplitude, inappropriate parameters, and unexplained image loss during the image acquisition process. Meanwhile, bladder volumes exceeding 30% compared to the planning CT images were excluded.

Consequently, a total of 303 iCBCT scans were analyzed, with an average number of scans per patient of 20.2 (ranging from 13 to 28 scans).

### CTV volume changes

We observed differences in the decrease in the CTV-U and CTV-C volume on iCBCT images after treatment (Table 2). The mean pretreatment and posttreatment volumes of the CTV-U and CTV-C were 58.9 cc and 43.4 cc, and 123.4 cc and 66.3 cc, respectively. The mean change in the CTV-U and CTV-C volume was 20.3% and 44.3%, respectively, with a larger change observed in the CTV-C volume (*P* < 0.001).

**Table 2:** Pretreatment vs. posttreatment clinical target volumes of the uterus and cervix on iCBCT images

Patients	Pretreatment volume of CTV-U (cc)	Posttreatment volume of CTV-U (cc)	Reduction in CTV-U volume %	Pretreatment volume of CTV-C (cc)	Posttreatment volume of CTV-C (cc)	Reduction in CTV-C volume %
1	122.1	75.6	38.1	225.3	93.1	58.7
2	58.9	54.6	7.3	165.4	139.2	15.8
3	33.5	30.3	10.6	87.9	46.2	47.4
4	28.3	27.1	4.2	83.6	77.7	7.1
5	35.6	32.6	8.4	77.1	48.6	37.0
6	23.9	13.4	43.9	111.8	41.2	63.1
7	18.7	18.3	2.1	51.6	29.8	42.2
8	42.3	40.3	4.7	106.0	57.4	45.8
9	16.9	14.6	13.6	79.1	46.6	41.1
10	59.4	35.2	40.7	111.1	43.4	60.9
11	161.4	78.5	51.4	225.3	93.1	58.7
12	48.3	32.1	33.5	93.4	53.7	42.5
13	105.9	89.6	15.4	198.3	99.1	50.0
14	68.9	59.9	13.1	135.4	77.6	42.7
15	59.4	49.2	17.2	99.2	48.4	51.2
Mean	58.9	43.4	20.3	123.4	66.3	44.3

Abbreviations: CTV-U, clinical target volumes of the uterus; CTV-C, clinical target volumes of the cervix; iCBCT, iterative cone beam computed tomography

### Evaluation of CTV-U and CTV-C motion

**Fig. 1:**
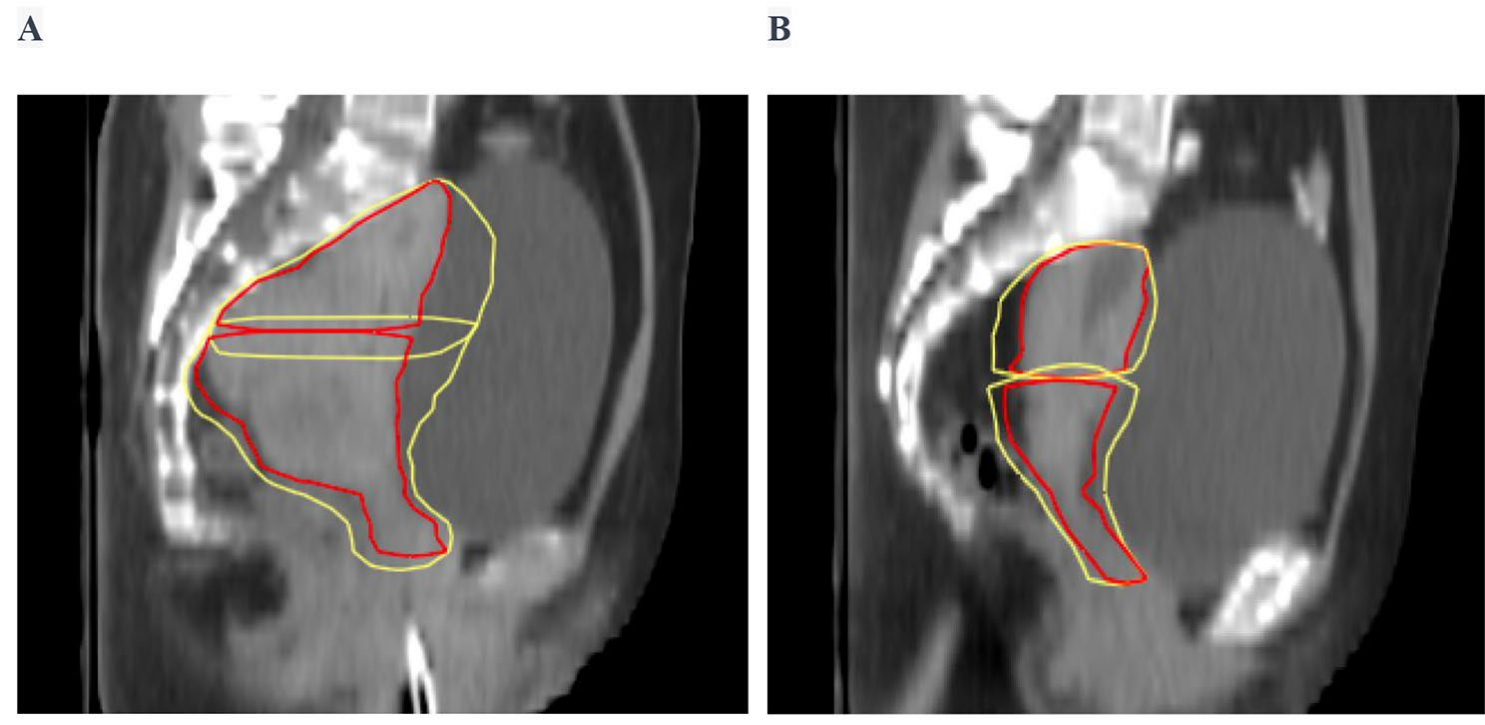
Sagittal slices of a planning CT scan from different patients. Daily clinical target volume contours from the iCBCT were mapped to the planning CT after registration with respect to bony anatomy. Initial CTV-U and CTV-C are shown in red, ultimate CTV-U and CTV-C are shown in yellow

Figure 1 depicts the fluctuations in motion for CTV-U and CTV-C by superimposing their contours onto the planning CT scans of various patients. The contours for the ultimate CTV-U and CTV-C are highlighted in yellow, while the initial CTV-U and CTV-C on the planning CT are presented in red. Sagittal images reveal instances where the initial CTV-U and CTV-C were sufficiently covered by the respective ultimate CTV-U and CTV-C. Large inter-fractional movements of the uterus and cervix for patient A occurred in the anterior direction. Patient B had a similar increase in the anterior and posterior margins of the CTV in the fundus and cervix during the treatment.

**Fig. 2:**
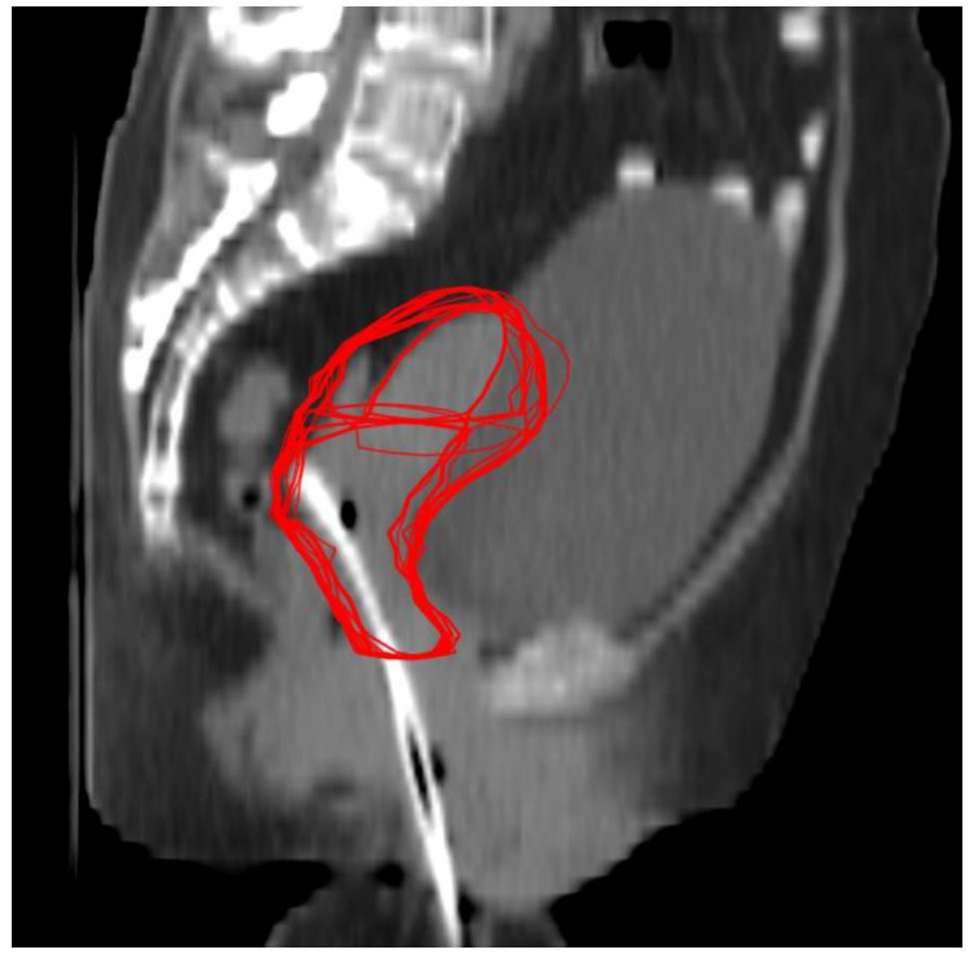
Sagittal image taken from a planning CT scan of one patient. All the CTV-Us and CTV-Cs (red) contours from the iCBCT were mapped to the planning CT after rigid registration with respect to bony anatomy

**Fig. 3:**
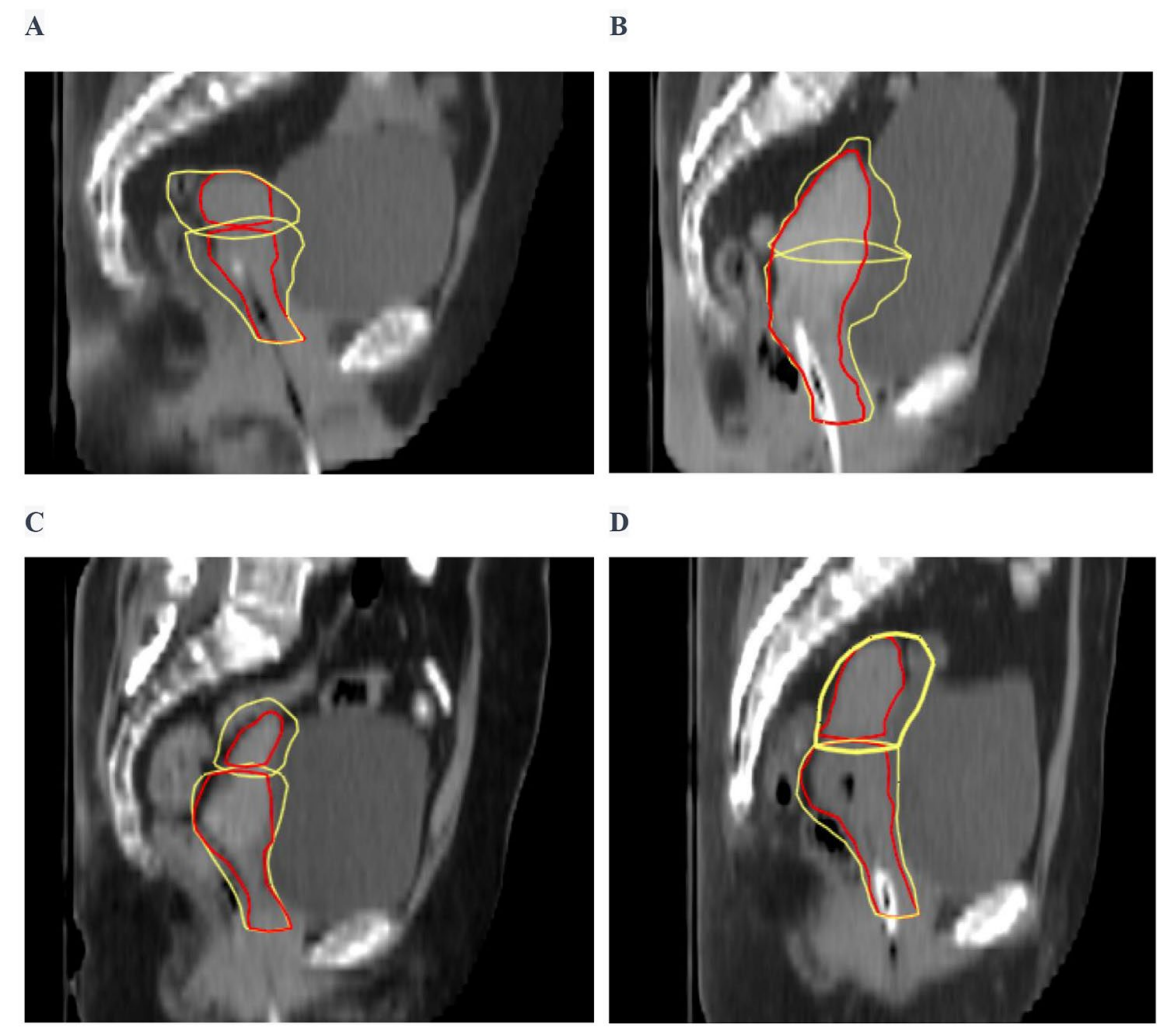
(**A**) Sagittal image of a planning CT scan from cervical cancer with stage T1. (**B**) Sagittal image of a planning CT scan from cervical cancer with stage T2. (**C**) Sagittal image of a planning CT scan from cervical cancer with stage T3. (**D**) Sagittal image of a planning CT scan from cervical cancer with stage T4. Initial CTV-U and CTV-C are shown in red, ultimate CTV-U and CTV-C are shown in yellow

Figure 2 depicts all the CTV-Us and CTV-Cs from the iCBCT (in red) of a patient mapped onto the planning CT. The average motion of the CTV-U in the left, right, anterior, posterior, superior, and inferior directions was 8.3 ± 4.1 mm, 9.8 ± 4.4 mm, 12.6 ± 4.0 mm, 8.8 ± 5.1 mm, 5.7 ± 5.4 mm, and 3.0 ± 3.2 mm, respectively. For the CTV-C, the average motion in the left, right, anterior, posterior, superior, and inferior directions was 7.3 ± 3.2 mm, 8.6 ± 3.8 mm, 9.0 ± 6.1 mm, 8.4 ± 3.6 mm, 5.0 ± 5.0 mm, and 3.0 ± 2.5 mm, respectively. To understand whether the changes in the CTV-U and CTV-C positions were affected by the tumor (T) stage, we correlated motions in the CTV-U and CTV-C positions with different T stages (Fig. 3). As shown in Table 3, the motion of CTV-U and CTV-C varies based on different T stages; in stage T1 patients, both the CTV-C and CTV-U showed greater motion in the AP and LR directions.

**Table 3:** The motions of CTV-U and CTV-C with different tumor stages

	T stage	Left	Right	Anterior	Posterior	Superior	Inferior
**CTV-U**	T1	12.8 ± 3.8	12.7 ± 0.9	15.6 ± 4.1	16.7 ± 1.2	5.0 ± 4.1	3.3 ± 2.4
	T2	8.2 ± 3.7	8.3 ± 3.3	13.7 ± 2.8	7.2 ± 3.6	6.4 ± 6.4	3.6 ± 3.5
	T3	6.2 ± 0.3	8.9 ± 3.8	8.1 ± 1.8	6.6 ± 2.4	3.3 ± 2.4	1.7 ± 2.4
	T4	5.5 ± 0.7	12.3 ± 5.6	11.1 ± 5.5	6.3 ± 0.9	7.5 ± 2.5	2.5 ± 2.5
**CTV-C**	T1	12.6 ± 0.7	12.2 ± 2.4	10.0 ± 1.8	12.8 ± 0.9	3.3 ± 2.4	1.7 ± 2.4
	T2	6.7 ± 3.6	7.4 ± 2.7	9.7 ± 7.7	7.8 ± 3.2	5.7 ± 5.6	4.3 ± 1.7
	T3	4.9 ± 0.2	8.9 ± 5.3	6.8 ± 1.4	5.8 ± 1.2	3.3 ± 2.4	1.7 ± 2.4
	T4	5.5 ± 0.8	7.3 ± 0.2	7.9 ± 2.0	8.0 ± 2.7	7.5 ± 2.5	2.5 ± 2.5

Abbreviations: CTV-U, clinical target volumes of the uterus; CTV-C, clinical target volumes of the cervix, T, tumor

### Target coverage and planning margins

As shown in Table 4, uniform margins > 25 mm would be required to encompass the entire CTV-U and CTV-C for every fraction. The movements of the cervix and uterine corpus were different. Uniform margins > 25 mm for the CTV-C and > 20 mm for the CTV-U would be required to encompass the two different CTVs for every fraction. With a 15 mm expansion margin in all directions, 88.9% of the CTV-U can be encompassed, while 97.8% of the CTV-C can be covered. For CTV-U, a 15-mm margin would miss 13%, 7%, 27%, and 13% of patients in the left, right, anterior and posterior directions, respectively. For CTV-C, a 15-mm margin would only miss 7% and 7% of patients in the right and anterior directions, respectively. A 15-mm margin, on average, would encompass more of the CTV-C volume.

**Table 4:** Differernt margins required to encompass the clinical target volume of uterus and cevix

	Marigin	Mean % of patients missing(Left)	Mean % of patients missing(Right)	Mean % of patients missing(Anterior)	Mean % of patients missing (Posterior)	Mean % of patients missing (Superior)	Mean % of patients missing (Inferior)
**CTV-U**	3	100	100	100	87	67	53
	5	87	80	100	80	33	7
	7	53	67	87	60	33	7
	10	20	53	80	27	13	0
	15	13	7	27	13	0	0
	20	0	0	7	0	0	0
	25	0	0	0	0	0	0
	30	0	0	0	0	0	0
**CTV-C**	3	93	100	93	100	67	60
	5	60	80	80	87	27	0
	7	33	67	60	60	27	0
	10	33	27	33	33	7	0
	15	0	7	7	0	0	0
	20	0	0	7	0	0	0
	25	0	0	7	0	0	0
	30	0	0	0	0	0	0

Abbreviations: CTV-U, clinical target volumes of the uterus; CTV-C, clinical target volumes of the cervix

We then sought to estimate the volume of the CTVs when planning CT before and after treatment. Compared with the initial CTV-U before treatment, the average percentage and volume increase were 160% (range, 52–393%) and 66 cc (range, 28–120 cc), respectively. Compared with the initial CTV-C before treatment, the average percentage and volume increase were 51% (range, 26–157%) and 59 cc (range, 23–151 cc), respectively. A statistically significant difference in the increase in the percentage of the CTV-U in comparison to the CTV-C was observed (*P* < 0.001), which implies that the motion of the CTV-U was larger during RT.

## Discussion

Precise target localization is an important goal in the treatment of cervical cancer. Uncertainties may stem from both inter- and intra-fractional motion, with inter-fractional motion potentially exerting a more significant influence [14]. Inter-fractional motion can occur due to the tumor and surrounding organ movements, deformations, and volume changes; therefore, to ensure high precision, the rational application of margins to the CTV is a crucial approach [8]. Meanwhile, IMRT is an essential tool for effectively addressing and minimizing uncertainties during treatment [15].

In this study, we evaluated the PTV motion during RT for cervical cancer with daily iCBCT. Previous studies have paid more attention to the uniform margin of the CTV [9]. Our study found that the motions of the target volume were different in six directions. The change in the CTV-C volume was larger than that of the CTV-U. According to the T stage, the CTV-C and CTV-U of patients with stage T1 showed greater motion. In addition, the results showed that the movements of the cervix and uterine corpus were different. A uniform 15-mm margin would have missed some of the CTV-U and CTV-C volumes. Thus, personal margins during RT for cervical cancer are necessary.

Different image-guided systems for RT have been applied in the study of the ITV for cervical cancer, including magnetic resonance imaging (MRI) and cone beam computed tomography (CBCT) [16]. Chan et al. [14] reported the movement of the external cervix oscillated between 10 mm and 15 mm during EBRT with weekly MRI scans, suggesting the utilization of daily images to maximize the advantages of high-precision radiation in patients with cervical cancer. Langerak et al. [17] conducted a comparison between the planning CT scans of cervical cancer and daily CBCT to identify variations. They observed mean shifts of 0.4 mm, 1.0 mm, and − 3.9 mm in the RL, AP, and SI directions, respectively. However, the CBCT images exhibited suboptimal quality, which was attributed to scattering and artifacts [18]. As a technological advancement, iCBCT attains uniform imaging with reduced noise and enhanced quality [19]. To our knowledge, there is limited research using iCBCT images to investigate the motion of the target area in cervical cancer.

In addition, several studies have focused on the movements of the uterine corpus and cervix. The average displacements documented for the uterine corpus varied between 3.3 mm and 14.2 mm in the AP direction, 6.1 mm to 9.5 mm in the SI direction, and 0.7 mm to 6.5 mm laterally [20]. As for the cervix, the amplitude of the mean inter-fractional movements ranged from 2.4 mm to 16 mm in the AP direction, 1.5 mm to 8 mm in the SI direction, and 0.3 mm to 10 mm laterally [21]. Taylor et al. [22] demonstrated that the uterus is prone to significant displacements whereas cervical movement is comparatively less pronounced. This is similar to the results of our study. Due to bladder and rectum filling, large movements occur in the SI and AP directions compared to the LR direction [23]. Researchers propose an asymmetrical margin with the CTV–PTV margin of the uterus and cervix [22]; however, the disadvantage of this strategy was insufficient target coverage [24]. Therefore, further research is required.

Tumor regression has the potential to significantly impact the positioning of organs and structures during RT for cervical cancer [20, 25]. Beth et al. [7] investigated tumor regression and cervical mobility in the context of concurrent chemoradiotherapy for cervical cancer. Their study revealed that the average cervical volumes before and after receiving 45 Gy of EBRT were 97.0 cc and 31.9 cc, respectively, indicating a mean volume reduction of 62.3%. The regression observed in their research resulted in a change in the position of the cervix. In our study, the mean reduction in the CTV-C and CTV-U volumes on iCBCT images was 44.3% and 20.3%, respectively. Notably, the mean change in the CTV-C volume was larger than that for the CTV-U. In addition to tumor regression, the T stage of the tumor may exert an influence on the mobility of the target area. To our knowledge, this study is the first to use daily iCBCT imaging to evaluate the relationship between changes in the target and tumor stage. Different T stages of the tumor represent the relationship between the lesion and surrounding organs [26]. In patients with stage T1, the variation in the CTV-C and CTV-U was greater, especially in the AP and LR directions. One reason may be that the carcinoma is strictly confined to the cervix, rectum, and bladder in stage T1, having a significant impact on the target volume [27].

Some studies involving patients with cervical cancer have explored the inter-fractional motion of the cervix using imaging modalities such as computed tomography, MRI, and portal imaging with fiducial markers [14, 28, 29]. Motion was assessed either directly at the cervix (center of mass, cervical boundaries) or by employing fiducial markers as a surrogate for cervical motion [21]. We developed a novel procedure using Python to automatically analyze the daily iCBCT images and understand the boundaries of the CTV expansion. In comparison to previous studies, our research offers a more convenient and accurate means of assessing the mobility of the CTV in cervical cancer.

The requirement for uniform large margins to accommodate the movement of the target volume results in acute gastrointestinal and genitourinary toxicity [30]. Applying asymmetric planning margins around subregions of the CTV is a potential approach to reducing margins [9, 31]. A reduction in margin implies a decreased dose of the OARs, and the incidence of adverse reactions exhibited a downward trend [32]. Advancements in technology have led to the implementation of adaptive radiotherapy (ART), which could tailor treatment to these changes and provide more benefits [31]. Previous studies have shown the dosimetric benefits of reduced margins in comparison to larger conventional margins through daily ART in pelvic cancers [33]. Meanwhile, Wang et al. [32] found that PTV margins could reduce to 5 mm by daily online ART, which significantly decreases the dose to critical organs at risk and potentially lead to a lower incidence of acute toxicity in cervical cancer.

Nevertheless, this study has several limitations. While our study entailed a meticulous analysis of a substantial volume of iCBCT scans, the patient sample size was limited, leading to corresponding imprecision in our estimates. In addition, the disadvantage of iCBCT is poor soft tissue display, which has some influence on contouring. Finally, our novel program can be further confirmed by more data analysis.

## Conclusions

Margining algorithms, in combination with daily iCBCT, can obtain the motion of the CTV-C and CTV-U during the treatment of cervical cancer. The movements of the uterine corpus are larger than those of the cervix. Early T stages require larger margins. Tailored margins present potential solutions for optimizing target volume coverage while minimizing the dose to OAR. Together, these findings suggest that the strategy we report could improve the understanding of the RT requirements for patients with cervical cancer. Validation of these findings with clinical outcomes is essential before their integration into routine clinical practice.

## Data Availability

The raw data supporting the conclusions of this article will be made available by the authors, without undue reservation.

## Abbreviations

AP: Anterior-Posterior
ART: Adaptive radiotherapy
CBCT: Cone beam computed tomography
CTV: Clinical target volume
CTV-C: Clinical target volumes of the cervix
CTV-U: Clinical target volumes of the uterus
iCBCT: Iterative cone beam computed tomography
IMRT: Intensity-modulated radiation therapy
ITV: Internal target volume
LR: Left-Right
MRI: Magnetic resonance imaging
OARs: Organs at risk
PTV: Planning treatment volume
RT: Radiation therapy
SI: Superior-Inferior
T: Tumor
